# Safety and efficacy of cannabidiol-cannabidiolic acid rich hemp extract in the treatment of refractory epileptic seizures in dogs

**DOI:** 10.3389/fvets.2022.939966

**Published:** 2022-07-29

**Authors:** Gabriel A. Garcia, Stephanie Kube, Sheila Carrera-Justiz, David Tittle, Joseph J. Wakshlag

**Affiliations:** ^1^Department of Clinical Sciences, University of Florida College of Veterinary Medicine, Gainesville, FL, United States; ^2^Veterinary Neurology and Pain Management Center of New England, Walpole, MA, United States; ^3^Ellevet Sciences, South Portland, ME, United States

**Keywords:** dog, seizure, cannabidiol, cannabidiolic acid, phenobarbital, zonisamide

## Abstract

The use of cannabidiol (CBD) in childhood refractory seizures has become a common therapeutic approach for specific seizure disorders in human medicine. Similarly, there is an interest in using CBD, cannabidiolic acid (CBDA) or cannabinoid-rich hemp products in the treatment of idiopathic epilepsy in dogs. We aimed to examine a small cohort in a pilot investigation using a CBD and CBDA-rich hemp product for the treatment of refractory epileptic seizures in dogs. Fourteen dogs were examined in a 24-week randomized cross-over study being provided placebo or CBD/CBDA-rich hemp extract treatment at 2 mg/kg orally every 12 h for each 12-week arm of the study. Serum chemistry, complete blood counts, serum anti-seizure medication (ASM) concentrations and epileptic seizure frequency were followed over both arms of the cross-over trial. Results demonstrated that besides a mild increase in alkaline phosphatase, there were no alterations observed on routine bloodwork at 2, 6, and 12 weeks during either arm of the study. Epileptic seizure frequency decreased across the population from a mean of 8.0 ± 4.8 during placebo treatment to 5.0 ± 3.6 with CBD/CBDA-rich hemp extract (*P* = 0.02). In addition, epileptic seizure event days over the 12 weeks of CBD/CBDA-rich hemp treatment were 4.1 ± 3.4, which was significantly different than during the 12 weeks of placebo treatment (5.8 ± 3.1; *P* =0.02). The number of dogs with a 50% reduction in epileptic activity while on treatment were 6/14, whereas 0/14 had reductions of 50% or greater while on the placebo (*P* = 0.02). No differences were observed in serum zonisamide, phenobarbital or bromide concentrations while on the treatment across groups. Adverse events were minimal, but included somnolence (3/14) and transient increases in ataxia (4/14) during CBD/CBDA-rich hemp extract treatment; this was not significantly different from placebo. This further indicates that providing CBD/CBDA-rich hemp extract during refractory epilepsy (only partially responsive to ASM), in conjunction with other ASM appears safe. Based on this information, the use of 2 mg/kg every 12 h of a CBD/CBDA-rich hemp extract can have benefits in reducing the incidence of epileptic seizures, when used concurrently with other ASMs.

## Introduction

Since the 1970's or earlier, cannabinoids have been found to have anti-epileptic effects in animal seizure models (1). These anti-epileptic findings also show that the effects of cannabinoids were separate from the psychotropic and excitatory effects of Δ9-tetrahydrocannabidiol (THC) and that CBD exhibited a lack of central nervous system excitation (1, 2). Following the approval of a highly purified CBD formulation, licensed in humans^1^ specifically for the treatment of seizures associated with Dravet Syndrome, Lennox-Gastaut Syndrome and more recently Tuberous Sclerosis Complex, there has been a resurgent interest in the use of CBD for various forms of epileptic activity in various species (3–5).

In their review article, Franco et al. summate that no one mechanism appears to be solely responsible for the anti-epileptic action of CBD (6). Goerl et al. explored the growing evidence that CBD alone does not solely contribute to the anti-epileptic properties seen in the use of such preparations (4). They concluded from their study that hemp extracts containing increased levels of CBDA also demonstrated similar anticonvulsant activity in a mouse epilepsy model; the presence of minor cannabinoids alongside CBDA augmented potency, providing further conceptual evidence of the “entourage effect” (4).

The pharmacokinetics, safety and efficacy of a blend of a CBD/CBDA-rich hemp extract in osteoarthritic dogs at 2 mg/kg in an oil base provided orally every 12 h, have been previously demonstrated (7). Previous work describes CBD and CBD/CBDA-rich hemp extract administration being safe at doses between 2 and 10 mg/kg body weight provided as an orally administered oil (8, 9). Other studies have found mild adverse events associated with the administration of CBD to dogs; these gastrointestinal signs were attributed to the medium-chain triglyceride oil base of the product rather than the cannabinoids (3).

Various studies in both humans and animals, have demonstrated epileptic seizure reduction following administration of CBD alongside commonly prescribed ASM (10, 11). Furthermore, McGrath et al. found a significant, thirty-three percent reduction in epileptic seizure days (seizure days being defined as one or more events during the day to account for cluster seizures) in dogs administered CBD, compared to baseline reported seizure events. Two of nine dogs in the treatment group showed a fifty percent or greater reduction in epileptic seizure events (11).

Drug-to-drug interactions between various ASM and CBD have been explored in numerous studies and review articles in both humans and animals (3, 6, 10–12). CBD is metabolized in the liver and the gastrointestinal tract, *via* cytochrome P450 (CYP) isoenzymes (13). These enzymes are also involved in the metabolism of many ASMs, and so some interaction or inhibition of metabolism could be anticipated (14). Conversely, McGrath et al. found no significant difference in serum phenobarbital or bromide concentration with concurrent CBD administration, during their study period (11). Additionally, Gaston et al. did report interaction between some commonly used ASMs and CBD in human studies with increased serum concentrations of topiramate and zonisamide, although no information is currently available in veterinary medicine (14). No effects on serum concentrations of phenobarbital, levetiracetam, or pregabalin in humans have been noted (14). This concurs with Doran et al. who found no variation in canine phenobarbital serum concentration with co-administration of CBD-rich hemp, further clarifying that CBD-rich hemp when treating concomitantly with phenobarbital may not need to be monitored more frequent during co-administration (3).

Dose-related somnolence and sedation is a commonly reported side effect of CBD administration (10, 14) and is of greater magnitude in human patients receiving the ASM, clobazam. McGrath et al. found no evidence of increased sedation in their canine study cohort; however there were 2 dogs that discontinued the study due to ataxia in the CBD-rich hemp group (11).

The aim of this study was two-fold: (1) determine whether CBD/CBDA rich hemp extract can help control canine refractory epileptic seizure patients who are only partially responsive to commonly utilized ASMs and (2) determine the adverse events of this treatment through owner survey, physical examination, serum biochemistry and ASM serum concentration evaluation during treatment in a 3 month cross over blinded clinical trial.

## Materials and methods

### Study participants

The protocol for clinical research was approved by the University of Florida Institutional Animal Care and Use Committee (UF-0056-2019) that included two study sites [University of Florida Small Animal Hospital (UFSAH) and the Veterinary Neurology and Pain Management Center of New England (VNPMCNE)] which began in September of 2019 and terminated in June of 2021. Prior to enrolment, all clients were provided with a client consent form outlining the study and the expectations during the research protocol.

#### Inclusion/exclusion criteria

All dogs initially enrolled (*n* = 10) in the study had prior magnetic resonance imaging (MRI) and cerebral spinal fluid (CSF) analysis for infectious or structural lesions. Dogs had complete blood counts, serum chemistry, pre and post-feeding bile acids, thyroid hormone assessment and urinalysis to rule out other metabolic issues as defined by the International Veterinary Task Force level II confidence (15). All dogs from this cohort had been treated for over a year and remained only partially responsive to common highest tolerable doses of ASM yet still having at least one epileptic event per month as criteria for enrolment (15). Dogs must have been on the current medications over the past 3 months with no new additions of ASM during the time, prior to enrolment. For all dogs, no new medications could be added throughout the study period, but alterations in phenobarbital or potassium bromide dosing could occur, based on adverse events reported by the owner (i.e., ataxia, excessive lethargy, or somnolence) or blood work abnormalities (i.e., outside or reference range when monitoring ASM, adverse hepatic, or renal parameter alterations). All dogs were required to be negative for infectious disease titers including all tick-borne diseases, toxoplasma and neospora, as well as *Dirofilaria Immitis*. All dogs involved in the study could not have endocrine issues or organ dysfunction, other than concomitant osteoarthritis issues and could be on non-steroidal anti-inflammatories and non-hemp based nutraceuticals, but owners were informed that the regimen could not be altered over the 6-month study period.

After initial enrolment, the enrolment criteria was modified to increase dog numbers as there were patients identified that had been on ASM for long duration (over 1 year) and showed no bloodwork or urinalysis abnormalities indicating metabolic issues, yet the owners were resistant to general anesthesia for MRI and CSF assessment and were enrolled based on level 1 confidence of the International Veterinary Epilepsy Task Force (15). Therefore, five dogs without MRI or CSF assessment with presumed idiopathic epilepsy were enrolled from one institution (UFSAH). The attending neurologist was confident were these dogs had signalments of at least a 1-year duration of refractory epileptic events (one ore more epileptic events per month) that were being managed on highest tolerable doses ASMs with only partial responses.

### Study design

This study was designed as a randomized placebo blinded cross over, where dogs were randomized into placebo or CBD/CBDA-rich hemp treatment for 3 months, using a random number generator (Randomizer Smartphone App); and then switched after 3 months with no washout period. Treatment consisted of a hemp derived cannabinoid therapy in sesame seed oil. The oil suspension was utilized to make 10, 25, and 50 mg capsules to be dispensed. Cannabinoid analysis of the product was ~30 mg/mL of CBD, 31 mg/mL of CBDA, 1.2 mg/mL THC, 1.3 mg/mL tetrahydrocannabinolic acid (THCA), 1 mg/mL of cannabichromene and 1.2 mg/mL of cannabichromic acid. Placebo was formulated similarly utilizing the same volume of sesame oil in comparable capsules. All placebo bottles were lined with a thin layer of cannabinoid depleted terpenes, to provide the same odor upon opening the bottle to aid in blinding owners to the treatment vs. placebo and were provided capsules that would equate to the same number of capsules that were provided from the treatment phase. For the CBD/CBDA-rich hemp extract oil, patients were dosed with variations in numbers of capsules as close to 2 mg/kg body weight every 12 h; medications were dispensed to provide enough supply to be sufficient between each 6 week study visit, so that medication residuals could be assessed for compliance.

### Complete blood counts, serum chemistry, and ASM concentrations

Dogs were re-evaluated at 2, 6, and 12 weeks of each arm of the cross-over study. At these time points, blood was drawn to evaluate the complete blood counts, serum biochemistry and serum ASMs, including zonisamide, phenobarbital and bromide between 10 a.m. and 4 p.m. after morning dosing before arrival to the clinic. Serum phenobarbital and bromide concentrations were evaluated at the University of Florida Veterinary Clinical Pathology Department, whilst serum zonisamide was evaluated at the Auburn University Veterinary Diagnostic Laboratory. A serum biochemistry including sodium, potassium, chloride, phosphorus, calcium, magnesium, albumin, total protein, globulin, cholesterol, glucose, alanine aminotransferase (ALT), aspartate amino transferase (AST), alkaline phosphatase (ALP), bilirubin, urea nitrogen and creatinine was evaluated at each time point. Complete blood counts including white blood cell, red blood cells, hematocrit, hemoglobin, neutrophil, lymphocyte, platelet, eosinophil, and basophil counts were performed at the University of Florida Veterinary Clinical Pathology Department.

### Owner adverse event and epileptic seizure diary

At the initial visit, owners were provided with a epileptic seizure diary to log events and cluster activity (i.e., a cluster of seizures on a single day which is reported as seizure days) including the date and time that the event was recognized and any interventions, such as diazepam or oral ASM dosing beyond the typical regimen. At the end of the 12-week period, the diary was given to the investigators and the owners were provided a second diary to utilize for the next 12-week period of the cross-over design. For each diary the total number of seizure events were tallied over the 3 month period and considering some dogs displayed cluster seizures as separate epileptic events over a period of time in a single day we also recorded seizure days similar to a prior study examining CBD-rich hemp use in dogs with idiopathic epilepsy (11). In addition, success of treatment as a reduction of 50% in seizure activity was also examined as an outcome that is considered clinically significant (11). At the end of each 12-week treatment period, the owners were provided with a survey that utilized a Likert scale for a series of questions related to adverse events including diarrhea/vomiting, appetite, thirst/urination, lethargy/somnolence, ataxia and anxiety behaviors. A similar scale was used related to seizure severity, seizure frequency and overall control of seizures during both 12-week periods of placebo and treatment (see Supplementary material).

### Statistical analysis and record screening

Power analysis was performed based on prior pilot work examining CBD use in dogs with a median of 4 seizures, that decreased to 2.7 over the duration of the study; a standard deviation estimate of 1.3 seizures per month resulted in a presumed population of 16 dogs necessary to establish significance (11). Initially candidate records were screened based on MRI and CSF analysis being negative for structural or inflammatory disease, at the UFSAH and the VNPCNE.

Eight dogs were identified based on records at UFSAH and solicited, of which only 6 were initially screened at baseline examination, while 5 dogs at VNPCNE were screened and enrolled. Of these 11 candidates originally enrolled, only 1 dog discontinued from protocol within the first week, due to compliance issues (dog resistant to medicating) while initially taking the treatment medication. All 10 remaining dogs completed the protocol by the summer of 2020. The recent pandemic has however, prevented adequate enrolment, alongside client hesitance to perform anesthesia for MRI and CSF collection. This led to increasing enrolment to 5 more dogs that had idiopathic refractory epilepsy with at least 1 epileptic seizure per month, between June of 2020 and June of 2021. All of these dogs had partial response to ASM for over 1-year duration, without confirmation by MRI and CSF, and no alterations in treatment protocols within the prior 3 months of treatment. Four of the 5 dogs were recruited and completed the study. The remaining dog completed the study, but it was later found that phenobarbital was instituted at the initiation of the study, so the data were removed from the analysis. Hence, 14 dogs completed the protocols with adequate data for analysis.

Statistical analysis was performed with a commercially available software package (JMP 11.0; Cary NC, USA). All data were assessed utilizing a Shapiro-Wilk test, and residual plots were examined to determine normality. When normality was rejected, the data was log transformed before analysis utilizing a mixed model analysis of variance. Cross-over study variables included in the model were: fixed effects of treatment, time, sequence of treatments, treatment × time, as well as random effects of the observation period, period nested within dog, time point nested within period nested within dog. Tukey's tests were performed *post-hoc* on any significant effects of time, treatment or time × treatment to assess differences. A *p*-value of < 0.05 was determined to be significant for all analyses.

Epileptic seizure incidence data from the diaries was collected and enumerated as a total quantity of seizures in a 12 week period of time, with cluster events counted as separate events for seizure frequency tabulation; and total number of seizure days (to account for cluster seizures as a daily event) were recorded for both placebo and CBD/CBDA-rich hemp treatment. The total seizure numbers and seizure days during each 3-month period were assessed for normality and a Student's *T*-Test was performed, with a *p*-value for significance set at *p* < 0.05. As ordinal data, the 3-month owner survey questions related to severity, frequency and overall seizure management for placebo vs. CBD/CBDA-rich hemp oil treatment periods was collected and assessed utilizing a non-parametric Wilcoxon signed-rank test, with a *p*-value set at *p* < 0.05. For adverse events (diarrhea, vomiting, ataxia, urination frequency, appetite, and somnolence/lethargy, anxiety) when a score over the 3 month period was shown to increase to a score of 4 or 5 suggesting an increase in adverse events, then it was classified as a patient with an adverse event; vs. scores of 3 or less which were classified as no adverse event. These data were assessed using a Fisher's exact test to determine if there were any significant differences, when comparing the placebo and CBD/CBDA-rich hemp oil treatment groups with a *p*-value set at *p* < 0.05. In addition, responders with a 50% reduction in epileptic seizure events and days of seizures were also evaluated using a Fisher's exact test to determine significance between placebo and CBD/CBDA-rich hemp treatments with a *p*-value set at *p* < 0.05.

## Results

### Animals and recruitment

Of the 14 dogs, the median age at enrolment was 6.3 years (range 2.9–9.7 years). All dogs were neutered and included 3 females and 11 males. The median body weight at enrolment was 31.3 kg (range 4.2–40.9 kg). Breeds of dogs represented in this study were: 6 mixed breeds, 2 Labrador Retrievers, 2 Labradoodles, 1 Siberian Husky, 1 Chihuahua, 1 Golden Retriever and 1 German Shepherd.

### Complete blood count and serum chemistry

Complete blood counts and serum chemistry evaluations were performed on all dogs at each time point except for one dog from the VNPCoNE location, whose bloodwork was not performed due to a logistical error related to shipment while on placebo at week 12. All other time points were evaluated in the mixed model analysis of variance. All data were normally distributed, except for AST, ALT, and ALP which were log-transformed before analysis. Complete blood counts showed no treatment effects for any cell type evaluated over the course of both placebo or CBD/CBDA-rich hemp extract treatments (Table 1). There was a time effect showing a significant rise in basophils at week 2 of both placebo and CBD/CBDA-rich hemp extract treatment (*P* < 0.05).

**Table 1:** Complete blood count means and standard deviations at baseline, 2, 6, and 12 weeks of CBD/CBDA-rich hemp oil vs. placebo (*n* = 14) in a randomized cross over clinical trial.

**Parameter (reference range)**		**Baseline**	**2 wk**	**6 wk**	**12 wk**	***P*** **– Tx**	***P*** **– Time**	***P*** Tx*time
WBC (K/uL)	Placebo	8.71 ± 2.29	8.71 ± 1.90	8.95 ± 2.08	8.81 ± 2.17	0.68	0.74	0.86
	Treatment		9.00 ± 2.51	8.92 ± 2.84	9.21 ± 2.63			
RBC (M/uL)	Placebo	6.56 ± 0.69	6.65 ± 0.81	6.51 ± 0.74	6.64 ± 1.17	0.75	0.64	0.13
	Treatment		6.42 ± 0.78	6.66 ± 0.82	6.48 ± 0.81			
Hemoglobin (g/dL)	Placebo	16.2 ± 1.7	16.6 ± 1.7	18.6 ± 8.1	16.1 ± 2.12	0.41	0.36	0.57
	Treatment		16.0 ± 1.8	16.6 ± 1.8	16.6 ± 1.9			
Hematocrit (%)	Placebo	47.1 ± 4.8	48.1 ± 4.9	47.7 ± 4.2	47.1 ± 6.6	0.33	0.6	0.29
	Treatment		46.1 ± 5.1	48.1 ± 5.4	46.7 ± 4.8			
Platelet (K/uL)	Placebo	370 ± 132	389 ± 129	413 ± 141	408 ± 161	0.1	0.61	0.66
	Treatment		385 ± 146	373 ± 128	366 ± 115			
Neutrophil (K/uL)	Placebo	5.57 ± 1.69	5.58 ± 1.76	5.84 ± 1.66	5.66 ± 2.22	0.04	0.82	0.80
	Treatment		5.95 ± 2.23	5.91 ± 2.59	5.79 ± 1.59			
Lymphocyte (K/uL)	Placebo	1.84 ± 0.72	1.91 ± 0.67	1.92 ± 0.72	1.84 ± 0.60	0.02	0.96	0.96
	Treatment		1.81 ± 0.79	1.75 ± 0.65	1.81 ± 0.73			
Monocyte (K/uL)	Placebo	0.41 ± 0.24	0.42 ± 0.20	0.39 ± 0.16	0.46 ± 0.20	0.51	0.29	0.31
	Treatment		0.47 ± 0.19	0.44 ± 0.25	0.43 ± 0.22			
Eosinophil (K/uL)	Placebo	0.88 ± 0.70	0.74 ± 0.47	0.78 ± 0.51	0.81 ± 0.62	0.15	0.37	0.48
	Treatment		0.72 ± 0.56	0.77 ± 0.53	1.10 ± 1.22			
Basophil (K/uL)	Placebo	0.02 ± 0.01	0.04 ± 0.04*	0.02 ± 0.01	0.02 ± 0.01	0.61	<0.01	0.84
	Treatment		0.05 ± 0.08*	0.02 ± 0.02	0.02 ± 0.02			

*P-values for treatment, time and treatment over time show no treatment effects*.

**Figure 1:**
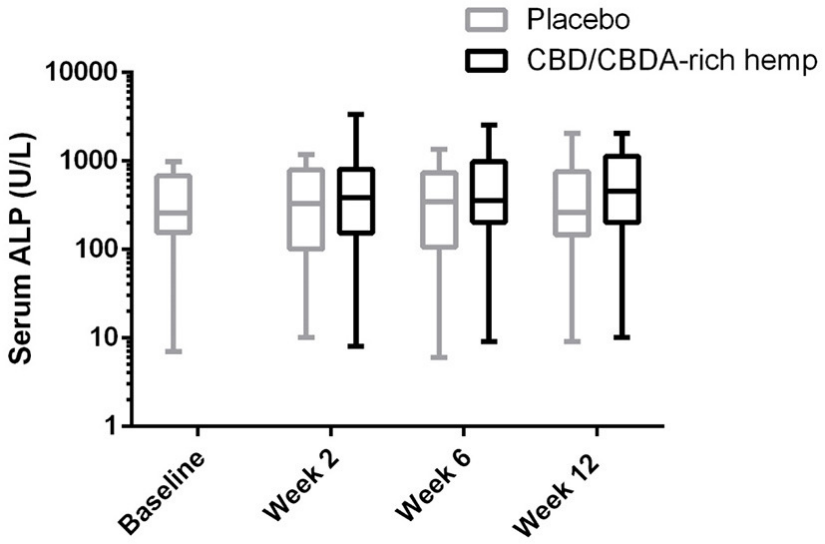
Log serum ALP activity shown over 12 weeks in both CBD/CBDA-rich hemp oil and placebo treatment phases of the study. No differences were observed across time or between groups at any time point, however there is a global treatment effect with slightly elevated ALP concentrations (*P* < 0.01).

Serum chemistry evaluations showed no significant changes for any serum electrolyte (sodium, potassium, chloride, phosphorus, calcium, or magnesium) over time or treatment (data not shown). Hepatic parameters (AST, ALP, bilirubin, cholesterol, glucose, albumin, and total protein) showed no significant alterations over time with treatment. Although no alterations in ALP could be deduced statistically over time between the groups there was a global treatment effect for ALP concentrations (Table 2; Figure 1). Renal parameters (creatinine, urea nitrogen) showed no significant changes over time or with treatment (Table 2).

**Table 2:** Serum chemistry hepatic and renal parameter means and standard deviations at baseline, 2, 6, and 12 weeks of CBD/CBDA-rich hemp oil vs. placebo treatment (*n* = 14) in a randomized cross over clinical trial.

**Parameter (ref/range)**		**Baseline**	**2 wk**	**6 wk**	**12 wk**	***P*** **– Tx**	***P*** **– Time**	***P*** – Tx*time
AST (U/L)	Placebo	28 ± 16	29 ± 15	30 ± 17	39 ± 34	0.63	0.63	0.59
	Treatment		30 ± 18	27 ± 15	26 ± 9			
ALT (U/L)	Placebo	76 ± 106	69 ± 72	70 ± 76	113 ± 166	0.50	0.95	0.78
	Treatment		63 ± 47	54 ± 37	43 ± 22			
ALP (U/L)	Placebo	362 ± 299	446 ± 377	465 ± 423	542 ± 577	<0.01	0.56	0.20
	Treatment		664 ± 879	707 ± 699	617 ± 561			
Bilirubin (mg/dL)	Placebo	0.2 ± 0.1	0.2 ± 0.1	0.2 ± 0.1	0.2 ± 0.1	0.13	0.90	0.98
	Treatment		0.2 ± 0.1	0.2 ± 0.1	0.2 ± 0.1			
Glucose (mg/dL)	Placebo	104 ± 13	99 ± 12	104 ± 31	103 ± 7	0.88	0.36	0.84
	Treatment		98 ± 9	98 ± 12	101 ± 12			
Cholesterol (mg/dL)	Placebo	234 ± 84	251 ± 90	229 ± 80	219 ± 54	0.96	0.25	0.64
	Treatment		242 ± 77	227 ± 63	256 ± 70			
Albumin (g/dL)	Placebo	2.8 ± 0.4	2.8 ± 0.4	2.9 ± 0.3	2.8 ± 0.4	0.41	0.45	0.28
	Treatment		2.8 ± 0.3	2.9 ± 0.3	2.8 ± 0.4			
Globulin (G/dL)	Placebo	2.8 ± 0.3	3.1 ± 0.4	3.1 ± 0.4	3.2 ± 0.5	0.75	0.29	0.18
	Treatment		3.0 ± 0.4	3.1 ± 0.4	3.1 ± 0.4			
Urea nitrog. (mg/dL)	Placebo	16.2 ± 2.6	16.8 ± 4.4	16.7 ± 4.0	16.8 ± 4.7	0.81	0.17	0.54
	Treatment		14.9 ± 5.8	18.1 ± 4.6	18.2 ± 3.1			
Total protein (g/dL)	Placebo	5.6 ± 0.5	5.9 ± 0.5	5.9 ± 0.5	6.0 ± 0.7	0.33	0.30	0.13
	Treatment		5.8 ± 0.5	6.0 ± 0.5	5.9 ± 0.4			
Creatinine (mg/dL)	Placebo	0.9 ± 0.2	1.0 ± 0.2	1.0 ± 0.3	0.9 ± 0.3	0.56	0.28	0.80
	Treatment		1.0 ± 0.3	1.0 ± 0.3	1.0 ± 0.2			

*P-values for treatment, time and treatment over time show no treatment effects except for ALP where a global treatment effect was noted*.

### Anti-seizure medication and serum concentrations

All dogs were on 3 or more ASMs consistently for 3 months before enrolment. Thirteen dogs were receiving potassium bromide; 11 dogs, zonisamide; 9 dogs were on phenobarbital; 13 on levetiracetam and one dog, topiramate (Supplementary Table 1). Alterations in medications were allowed during the protocol, yet few were observed. Potassium bromide was increased for one dog while on placebo and for one dog while on the CBD/CBDA-rich hemp oil. No dogs receiving zonisamide or levetiracetam had alterations in dosing throughout the 24-week study. Three dogs had phenobarbital decreased during the treatment phase, with one dog increasing the dose during the placebo phase, and one dog decreasing the dose during the placebo phase. One of the dogs receiving a lower dose during the treatment phase was maintained on the lower dose during the placebo phase.

Serum concentrations of ASMs were measured at each time point throughout the study. Serum zonisamide concentrations were assessed at all time points for 11 dogs, with the exception of 2 dogs, whose serum concentrations were not run at week 2. This was due to lack of serum for one dog; the second dog's serum was lost on shipment to the diagnostic laboratory. Serum zonisamide concentrations were not significantly different based on treatment or time, and no alterations due to treatment over time (Table 3). Serum phenobarbital concentrations were assessed on all 9 dogs being administered phenobarbital, except for one dog at week 12 during placebo treatment, due to logistical error regarding submission. Serum phenobarbital concentrations were not significantly different based on treatment, or over time and no alterations due to treatment over time (Table 3). Serum bromide concentrations were assessed on all 13 dogs being administered potassium bromide, except for one dog at week 12 during placebo treatment due to lack of serum, and week 2 for one dog due to serum loss during shipment to the diagnostic laboratory. Serum bromide concentrations were not significantly different based on treatment, or over time and no alterations due to treatment over time (Table 3).

**Table 3:** Serum concentrations of anti-seizure medications [ASM; potassium bromide (KBr), phenobarbital, zonisamide] that were co- administered with CBD/CBDA-rich hemp oil vs. placebo treatment measured at baseline, 2, 6, and 12 weeks.

**Serum ASM conc**.	**Treatment**	**Baseline**	**Week 2**	**Week 6**	**Week 12**	***P*** **– Tx**	***P*** **– Time**	***P*** – Tx*time
KBr (mg/mL)	Placebo	1.2 ± 0.6	1.4 ± 0.6	1.4 ± 0.6	1.3 ± 0.6	0.39	0.79	0.10
(*n* = 13)	Treatment		1.2 ± 0.6	1.3 ± 0.6	1.5 ± 0.5			
Phenobarbital (ug/dL)	Placebo	27.8 ± 7.8	30.1 ± 4.2	31.1 ± 3.2	29.0 ± 4.2	0.88	0.90	0.33
(*n* = 9)	Treatment		28.8 ± 4.9	27.7 ± 4.8	31.5 ± 7.2			
Zonisamide (ug/mL)	Placebo	35.8 ± 21.0	30.8 ± 12.7	36.4 ± 16.4	37.6 ± 17.3	0.83	0.77	0.37
(*n* = 11)	Treatment		38.4 ± 14.9	34.8 ± 14.3	36.3 ± 16.3			

*No significant differences were observed*.

### Seizure control assessment

**Figure 2:**
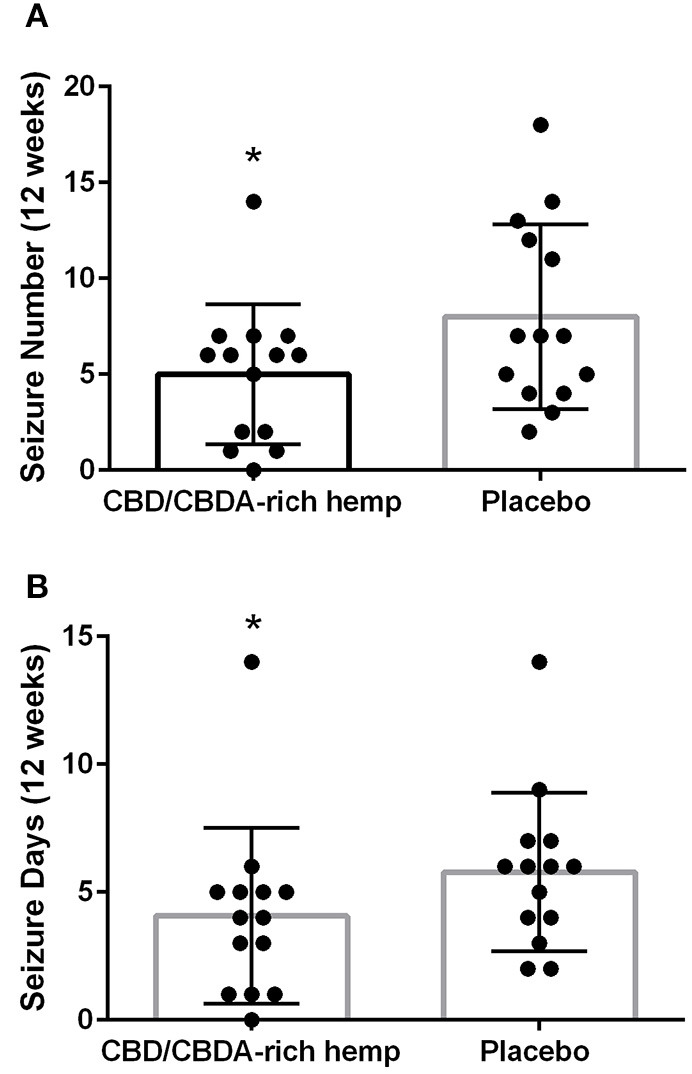
Mean seizure activity over 12-weeks of CBD/CBDA-rich hemp oil vs. placebo compared to placebo. **(A)** Epileptic seizure numbers recorded by owners during treatment using an encapsulated CBD/CBDA-rich hemp oil (2 mg/kg every 12 h) vs. placebo treatment. Asterisk indicates a significant difference (*P* = 0.02). **(B)** Total days of seizure activity over the 12 week period using an encapsulated CBD/CBDA-rich hemp oil vs. placebo treatment. Asterisk indicates a significant difference (*P* = 0.02).

Epileptic seizure events (as recorded by owners) were tallied over each 3-month period of time and the mean and standard deviations were calculated. Epileptic seizure events were all generalized in nature and no focal/partial seizures. The mean number of epileptic seizures during the placebo phase was 8.0 ± 4.8, while the mean number of seizures during the CBD/CBDA-rich hemp oil was 5.0 ± 3.6 (*P* = 0.02; Figure 2A). Mean epileptic seizure days in the control group over the 12-week period were 5.8 ± 3.1 and 4.1 ± 3.4 in the CBD/CBDA-rich hemp oil group (*P* = 0.02; Figure 2B). When assessed for a clinically significant 50% decrease in seizures between the two groups, the placebo group was 50% lower for 0 of 14 dogs, while 6 of 14 showed a 50% reduction in epileptic seizure events whilst on rich CBD/CBDA-rich hemp oil; this was statistically significant (*P* = 0.02). Similarly when assessing number of dogs with a 50% reduction in epileptic seizure days between groups, 0 of 14 in the placebo group showed a 50% reduction, while this level of reduction was seen in 5 of 14 in the CBD/CBDA-rich hemp oil group (*P* = 0.04).

### Owner assessments and adverse events

Owner Likert scores for seizure control and adverse events were collected for all respondents, except for one owner who did not provide the survey due to loss of contact at study end. The Likert scores were then compared between the two treatment phases for 13 of the 14 dogs enrolled. The average Likert score for owner perceived improvement in frequency of seizures was a median of 3 (no change; range 2–5) during the treatment phase. The median was 3 (no change; range 1–5) during the placebo phase, which was not significant (*P* = 0.41). Owner perception of improvement in severity for the CBD/CBDA-rich hemp oil treatment revealed a median value of 4 (a little improvement: range 2–5) and placebo median response was 3 (no change; range 1–5); this was not considered significant (*P* = 0.12). Further overall client assessment for overall epileptic seizure management revealed a median score of 4 (a little improvement; Range 2–5) while for placebo the median was 3 (no change; range 1–5) which showed no significant differences between the phases of treatment (*p* = 0.16).

Adverse events associated with increases in anxiety, vomiting or diarrhea, lethargy/somnolence, appetite, urination or thirst, and ataxia were assessed through Fisher's exact testing and found that for each of these potential adverse events associated with CBD/CBDA rich hemp vs. placebo, that there were no significant increases in these adverse events (Table 4).

**Table 4:** Adverse event report at end of 12 weeks of CBD/CBDA-rich hemp oil vs. placebo treatment.

**Adverse events**−**13**	**Placebo**	**Treatment**	**Fisher's**
**respondents**			**exact**
Increased appetite	0	3	*p* = 0.22
Increased GI signs (vomit/diarrhea)	0	2	*p* = 0.48
Increased thirst/urination	3	2	*p* = 1.0
Increased ataxia	1	4	*p* = 0.32
Increased anxiety	3	2	*p* = 1.0
Increased lethargy/somnolence	1	3	*p* = 0.59

*Fisher's exact testing showed no significant differences between groups*.

## Discussion

The major finding of our study is the statistically significant reduction in epileptic seizure frequency, as well as number of epileptic seizure days, as assessed through diaries kept by owners. Of course, unlike humans where precise accounts are likely, for dogs there may be seizures missed and unaccounted for. Usually, however, an episode can be determined by postictal activities or inappropriate soiling of surroundings, particularly since these were dog owners who were managing chronic epilepsy for over a year and were astute to behavioral changes. The epileptic seizure events in this population of dogs ranged from 3 to 14 seizures with a mean of 8 seizures over the 12-week study using placebo, which decreased to a mean of 5 seizures with a range of 0–12 events over the 12 weeks of treatment. A recent measure of successful treatment has been defined as a seizure free period of >3 times the longest pre-treatment interictal interval, which cannot be fully assessed as retrospective epileptic seizure diaries were not collected. Rather the epileptic seizure events during CBD/CBDA-rich hemp oil treatment was compared to the placebo treatment phase, whereby two dogs of the 14 dogs showed a 3-fold reduction in epileptic seizure events compared to placebo, as a full response (15). Another measure of partial success in the treatment of seizures is a 50% or greater reduction in seizure activity which is often reported in human literature and some veterinary literature (11, 16, 17). This would be defined as a partial response to an ASM (15), and is considered clinically significant. We found that a 50% reduction was observed in 6 of the 14 dogs during CBD/CBDA-rich hemp oil vs. placebo. If we examine dogs on CBD/CBDA-rich hemp oil treatment for both a 50% reduction in epileptic seizure frequency and epileptic seizure days ~40% of the patient population displayed minimally a partial response. These findings are very similar to the pilot study performed by McGrath et al. using a dose of 2.5 mg/kg of CBD oil orally every 12 h (11), while our study used a similar hemp extract dosing at ~1 mg/kg of CBD and 1 mg/kg of CBDA as an equal mix orally every 12 h.

CBD treatment has been well studied in both humans and rodents, showing reductions in seizure activity, particularly in various childhood conditions including Dravet's syndrome and Lennox- Gastaut syndrome (16–18). The current FDA approved CBD product (Epidiolex) is used at a dose of ~10–20 mg/kg, which is 10 times higher than doses that have now been utilized in dogs with partial responses. The difference in response between dogs may be related to the utilization of a whole hemp extract rich in CBD, vs. a purified CBD compound. Additionally, the formulation used in our study contains significant amounts of CBDA which has been shown to have anti-convulsant and anti-seizure activity in rodent models when delivered orally once per day (4); there is relatively sparse information regarding its use in humans, short of a pharmaceutical company trial suggesting efficacy of CBDA when provided orally once a day.^2^

CBDA is similar to CBD in its overall actions at neurological targets such as the glycine receptor, transient receptor potential villinoid and ankyrin receptors (TRPV, TRPA) and 5-hydroxytrapamine 1A receptors (5HT1A) with less known about CBDA's ability to interact with the equilibrative nucleoside transporter activity (19, 20). In humans, the pharmacokinetics of CBDA appear to be superior to CBD and have been examined in human clinical trials. Rodent model trials have shown that CBDA has similar efficacy in reducing seizure activity as CBD as a single molecule or blended as a 1:9 mixture of CBDA:CBD and taken at a total dose of 100 mg/kg (see text footnote 2). Mouse studies examining brain concentrations of CBDA suggest that it may not have the same nervous system penetration as CBD (4), due to the acidic form being less able to cross the blood brain barrier, while still displaying efficacy in a mouse model of Dravet Syndrome (21). Regardless, it seems to have similar activity and CBDA is known to be a superior agonist for the 5HT1A receptor (22, 23). The examination of CBDA as an anticonvulsant is in its infancy and further studies are necessary, yet it does seem to provide similar efficacy in dogs, to primary CBD-rich hemp treatment alone.

The whole hemp extract utilized in our study also contains small amounts of THC and THCA. THCA is the carboxylic acid form of THC which is found in the *Cannabis sativa* and is retained during extraction of the product used in this study. Primary preclinical rodent models have found benefit in providing THCA as a neuroprotective molecule for various degenerative diseases, including models of Parkinson's and Huntington's disease related pathologies (24, 25). THCA is non-psychotropic and sparsely studied regarding clinical investigation as a THC related molecule. Interestingly, prior examination of the pharmacokinetics in dogs has revealed that when equal small portions of THC and THCA are provided to dogs, there is a far more robust absorption of THCA than THC by ~10-fold (9). Serum concentrations when providing ~0.15 mg/kg of THCA reached between 20 and 30 ng/mL in the bloodstream while THC was ~2–5 ng/mL (9). The ability of THCA to reach the central nervous system has yet to be reported, but THCA appears to have variable effects on rodent seizure models and improvements in Huntington's disease models, as well as potent anti-inflammatory activity; this may be due to its interaction with the peroxisomal proliferation receptor system that modulates the inflammatory response (24, 25).

As a whole hemp extract, the cannabinoids may be able to act synergistically with the minor cannabinoids and other components (terpenes and flavonoids) to augment the response to the major cannabinoids CBD and CBDA. This is known as “the entourage effect” (26, 27). There is mounting evidence that constituent compounds within CBD-rich hemp extracts have efficacy as anti-epileptics and that dosing can be significantly decreased from CBD isolates (26, 27). A recent study examining children with refractory epilepsy showed that there were similar responses when using CBD-rich whole hemp extract with a CBD:THC ratio of ~20:1, at a total dose of ~5–6 mg/kg per day. Four of the 7 children examined had a 50% reduction in seizure frequency and all of these respondents displayed serum concentrations of CBD in the 15–25 ng/mL. When administered 10–12 mg/kg of cannabinoid rich hemp extract three of these 4 patients became seizure free, with steady state concentrations in the blood being between 52 and 124 ng/mL (26). A more recent meta-analysis of observational studies utilizing CBD-rich hemp products, as compared to purified CBD isolate products, showed similar response rates of ~35–40% with a 50% reduction in seizure frequency. The average daily dose administered was 6 mg/kg of CBD-rich hemp, vs. 25 mg/kg when using CBD isolate products. This further clarifies the validity of possible “entourage effects” observed when using whole hemp extracts, whereby a lower dose can be useful (27).

The use of CBD or CBD-rich hemp extracts can both lead to physiological changes; most notably an alteration in hepatic metabolism and increases in either ALT or ALP activity in dogs (7, 11). In a prior study examining the ALP activity over 12 weeks in dogs treated with a CBD-rich hemp extract for refractory epilepsy, a 2–4-fold increase in ALP serum enzyme activity was shown, suggesting potential alterations to hepatic drug metabolism (11). This rise can also be observed with ASMs like phenobarbital in dogs (28), hence examination of hepatic parameters are of utmost importance when using any ASM. Interestingly, we found no treatment over time effects when using CBD/CBDA-rich hemp extract, but there was a significant treatment effect overall suggesting mild to modest rises in ALP activity. It must be noted that 9 dogs in this study were receiving phenobarbital treatment which predisposed them to elevations; when examining the average increase, it was ~150–200 U/L which was suggestive of limited increases. Additionally, these alterations were within 2 weeks of initiating treatment and remained static for the 3 months of treatment. No other treatment effects were observed across serum chemistry or complete blood count assessment during the 12-week trial when compared to placebo.

In line with the ALP rise, there is expected to be some alterations in either induction or inhibition of the CYP enzyme system that is involved in the metabolism of drugs like phenobarbital and zonisamide (12, 14, 29, 30). In humans and rodents, the primary CYP known to be influenced by CBD administration are CYP3A4 and CYP2D19; these may not be identical in dogs considering the metabolites of CBD are dramatically different (29–32). This however, does not negate the need to understand whether CBD/CBDA-rich hemp alters the serum concentrations of these commonly prescribed ASM's. When measuring both phenobarbital and zonisamide concentrations over the 12-week period, there were no differences when compared to placebo treatment for 12 weeks. This suggests that CBD/CBDA-rich hemp does not affect the metabolism of these drugs or potassium bromide treatment when administered together. It must also be noted that two of the dogs did have increases in their potassium bromide dosing during the trial, one during the CBD/CBDA-rich hemp oil treatment phase and one during the placebo treatment phase, which can take up to 3 months to observe consistent changes in serum bromide concentrations. The small number of dogs needing alterations in their ASMs, and lack of this occurring in only one group, gives merit to the idea that CBD/CBDA-rich hemp treatment is not significantly affecting serum ASM concentrations. This lack of influence on serum ASMs concentrations is further corroborated in a recent publication by Doran et al., showing that neither CBD or phenobarbital concentrations differed when dogs were on 1 month of phenobarbital treatment, suggesting that these non-psychotropic cannabinoids can be administered together safely in epileptic dogs (3).

Owners were surveyed at the end of each 12-week phase of the study for improvements in severity, frequency and overall management of seizures. Surprisingly, owners did not find any improvement in frequency but did feel that the severity of seizures was improved on treatment. Overall management was slightly improved, with median Likert score improvements from a median of 3 for placebo to slightly improved (median score of 4) on treatment. The seizure frequency observed from diaries were likely influenced by recall bias, which is a factor that cannot be controlled in survey research. More importantly, no dogs were discontinued from the study due to adverse events related to the use of CBD/CBDA-rich hemp use over the 12-week period, other than an initial drop out due to difficulty in administering this particular product to the dog which may have been due to the typical hemp odor or the larger than usual number of capsules needed for this large dog. The results of adverse events when compared between groups assessed statistically showed no difference; however it must be noted that three owners reported somnolence and four reported a mild increase in ataxia while on the CBD/CBDA-rich hemp oil capsules. Increases in somnolence or lethargy was reported in only one dog while on the placebo capsules, and only one dog reported to be mildly more ataxic on the placebo. Other events, such as gastrointestinal disturbances were self-limiting and mild in nature with two dogs experiencing this during the CBD/CBDA-rich hemp oil treatment phase, and 3 dogs showing mild increases in appetite during the CBD/CBDA-rich oil treatment phase of the trial. Overall, the reported adverse events are comparable to that observed in larger human clinical trials regarding appetite, somnolence and ataxia (16–18). These are changes that should be monitored or discussed with clients interested in pursuing CBD-rich hemp treatment for epileptic seizure management (16–18).

A major limitation of any study with a small sample size of this nature is our inability to control consistent dosing; this is under the control of the client without confirmation of daily dosing. There was control to some extent by dispensing the initial 6 weeks of treatment or placebo and then asking about relative needs to complete the trial. This facilitated dispensing for the next few weeks, where owners did suggest that they were nearly out of capsules, allowing us to assume relatively regular twice daily dosing. Measuring serum cannabinoids at intervals would have allowed for better assessment of this factor and would have allowed for regression statistics to be run on the CBD serum concentrations to clinical response. McGrath et al. have suggested that responders in their study tended to have higher CBD levels on spot check after 12 weeks, inferring that there may be some benefit in monitoring serum CBD or other cannabinoid concentrations to better utilize CBD-rich hemp extracts clinically, much like other ASMs (11). At the time of initiating the study, there were no laboratories performing serum analysis of any cannabinoid other than CBD. Considering the product being tested contained other cannabinoids (namely CBDA) we felt that any assessment would be incomplete; therefore serum was not saved for future analysis. In addition, although this study suggests potential benefits for some dogs, the unique nature of this CBD/CBDA-rich hemp product cannot be extrapolated to other products. This is due to the unique nature of the CBD/CBDA blend, which is rare to find in the current cannabinoid nutraceutical market for dogs. The specific CBD/CBDA blend utilized in this study is readily available throughout the United States (Ellevet) with a current expansion project throughout the European Union, to include Greece, Germany, Netherlands, France and Spain. The same active product is freely available in the United Kingdom, marketed as a human preparation (Ellevance), due to current United Kingdom legislative restrictions on the provision of CBD based products to animals.

In conclusion, there appears to be a population of dogs that respond favorably to CBD/CBDA-rich hemp products for epileptic seizure reduction similar to other human, dog and rodent data. It must be noted that these benefits are observed as a multi-modal approach to ASM treatment and cannot be extrapolated to using CBD/CBDA-rich hemp as a single agent, due to a lack of studies. More importantly, the use of a CBD/CBDA-rich hemp does not appear to alter the hepatic metabolism of other ASMs, namely phenobarbital, zonisamide or potassium bromide during this 3 month period of treatment. Furthermore, the serum chemistry and complete blood count profiles across the 12-week trial period on treatment were no different than the placebo treatment phase, with the exception of a mild rise in serum alkaline phosphatase. Regardless, it is prudent to follow routine bloodwork to assess hepatic enzymes particularly when other ASMs, that are known to commonly cause increases in ALT and ALP concentrations, are being administered.

## Data availability statement

The raw data supporting the conclusions of this article will be made available by the authors, without undue reservation.

## Ethics statement

The animal study was reviewed and approved by University of Florida IACUC. Written informed consent was obtained from the owners for the participation of their animals in this study.

## Author contributions

GG and SK were the genesis of the idea, study design, data collection, initial analysis, and manuscript preparation/review. SC-J was involved in data collection, analysis, and manuscript preparation/review. DT and JW were involved in data analysis and manuscript review. All authors contributed to the article and approved the submitted version.

## Funding

GG received a grant from Ellevet Sciences to complete the clinical trial outlines in the manuscript and is an Advisory Board Member at Ellevet Sciences. Ellevet Sciences was not involved in the study design, collection, analysis, interpretation of data, the writing of this article or the decision to submit it for publication.

## Conflict of interest

Author GG received a grant from Ellevet Sciences to complete the clinical trial outlines in the manuscript and is an Advisory Board Member at Ellevet Sciences. Authors JW and DT are consultants at Ellevet Sciences and also serve as advisory board members at Ellevet Sciences. The remaining authors declare that the research was conducted in the absence of any commercial or financial relationships that could be construed as a potential conflict of interest.

## Publisher's note

All claims expressed in this article are solely those of the authors and do not necessarily represent those of their affiliated organizations, or those of the publisher, the editors and the reviewers. Any product that may be evaluated in this article, or claim that may be made by its manufacturer, is not guaranteed or endorsed by the publisher.

## Footnotes

^1^Epidiolex, Full (2020) Prescribing Information. Greenwich Biosciences Inc., Carlsbad https://www.epidiolex.com/sites/default/files/pdfs/VV-MED-03633_EPIDIOLEX_(Cannabidiol)_USPI.pdf (accessed November 23, 2021).

^2^GW Pharmaceuticals, World Intellectual Property Organization, Patents Number; WO2017/025712 A1. International Publication Date—February 16th, 2017.
