# Supplementary material for: Safety and efficacy of cannabidiol-cannabidiolic acid rich hemp extract in the treatment of refractory epileptic seizures in dogs
Source: Front Vet Sci. 2022 Jul 29;9:939966. doi: 10.3389/fvets.2022.939966 (PMC9372618; doi:10.3389/fvets.2022.939966)
Supplement: Supplementary file 1 [file Table_1.pdf]

Supplementary Data: CBD/CBDA-rich hemp trial survey

Date:_______________ **Owner Name:_____________________**

Visit (1-7): ____ **Dog Name:________________________**

Hemp based nutraceutical epilepsy and quality of life survey

Please answer these questions based on the last three months of treatment as it relates to adverse events, quality of life and seizure management.

Part A: For part A this scoring is based on the Likert scoring system which is:

1 = a lot more; 2 = a little more; 3 = no change; 4 = a bit less; 5 = a lot less.

In the past three months the clinical signs below regarding potential adverse events during treatment were:

1. Appetite:_________
2. Weight gain:_____________
3. Thirst/drinking:___________
4. Urination:_________
5. Lethargy/somnolence:____________
6. Ataxia (uncoordinated gait; “drunken when walking”):____________
7. Restlessness or anxiety:___________
8. Vomiting:_________
9. Diarrhea:_________

For Part B and C the Likert Scoring system is based on:

1 = strongly disagree; 2 = disagree; 3 = neutral; 4 = agree ; 5 = strongly agree.

B: Owners opinion about seizure severity and frequency

1. In the last three months the severity of seizures (duration of events) was acceptable:_______
2. In the last three months the frequency of seizures was acceptable:_________
3. In the last three months I found that overall the management of my dog’s seizures was acceptable: _____________

C: Quality of Life during treatment

1. In the past three months; the treatment with the nutraceutical has improved my dogs quality of life: ____________

Supplemental Table 1: ASM that dogs were taken prior to enrolment and any documented changes in medications during placebo or CBD/CBDA-rich hemp extract oil treatments.

Key: Pheno – phenobarbital; Zonis – zonisamide; Levi – levetiracetum; Topir - topiramate

DOG	Pheno	Zonis	Levi	KBr	Topir	Drug Δ placebo	Drug Δ CBD
1	No	Yes	Yes	No	No	None	None
2	No	Yes	Yes	Yes	No	None	None
3	Yes	No	Yes	Yes	No	Increase Pheno	Decrease Pheno
4	Yes	Yes	No	Yes	No	None	None
5	No	Yes	Yes	Yes	No	None	None
6	No	No	Yes	Yes	Yes	Decrease Pheno	Decrease Pheno
7	Yes	Yes	Yes	Yes	No	None	None
8	No	Yes	Yes	Yes	No	None	None
9	Yes	Yes	Yes	Yes	No	None	None
10	Yes	Yes	Yes	Yes	No	None	None
11	Yes	Yes	Yes	Yes	No	None	None
12	Yes	No	Yes	Yes	No	None	None
13	Yes	Yes	Yes	Yes	No	None	Increase KBr
14	Yes	Yes	Yes	Yes	No	Inc. KBR/Dec Pheno	Dec.Pheno
